# Sedimentary ancient DNA shows terrestrial plant richness continuously increased over the Holocene in northern Fennoscandia

**DOI:** 10.1126/sciadv.abf9557

**Published:** 2021-07-30

**Authors:** Dilli P. Rijal, Peter D. Heintzman, Youri Lammers, Nigel G. Yoccoz, Kelsey E. Lorberau, Iva Pitelkova, Tomasz Goslar, Francisco J. A. Murguzur, J. Sakari Salonen, Karin F. Helmens, Jostein Bakke, Mary E. Edwards, Torbjørn Alm, Kari Anne Bråthen, Antony G. Brown, Inger G. Alsos

**Affiliations:** 1 The Arctic University Museum of Norway, UiT The Arctic University of Norway, Tromsø, Norway.; 2 Department of Arctic and Marine Biology, UiT The Arctic University of Norway, Tromsø, Norway.; 3 Faculty of Physics, Adam Mickiewicz University, Poznań, Poland.; 4 Poznań Park of Science and Technology, Poznań, Poland.; 5 Department of Geosciences and Geography, University of Helsinki, Helsinki, Finland.; 6 Swedish Museum of Natural History, P.O. Box 50007, 10405 Stockholm, Sweden.; 7 Värriö Research Station, Institute for Atmospheric and Earth System Research INAR/Physics, University of Helsinki, P.O. Box 64, 00014 Helsinki, Finland.; 8 Department of Earth Science, University of Bergen, Bergen, Norway.; 9 School of Geography and Environmental Science, University of Southampton, Southampton, UK.; 10 Alaska Quaternary Center, University of Alaska, Fairbanks, AK 99775, USA.

## Abstract

The effects of climate change on species richness are debated but can be informed by the past. Here, we generated a sedimentary ancient DNA dataset covering 10 lakes and applied novel methods for data harmonization. We assessed the impact of Holocene climate changes and nutrients on terrestrial plant richness in northern Fennoscandia. We find that richness increased steeply during the rapidly warming Early Holocene. In contrast to findings from most pollen studies, we show that richness continued to increase thereafter, although the climate was stable, with richness and the regional species pool only stabilizing during the past three millennia. Furthermore, overall increases in richness were greater in catchments with higher soil nutrient availability. We suggest that richness will increase with ongoing warming, especially at localities with high nutrient availability and assuming that human activity remains low in the region, although lags of millennia may be expected.

## Teaser

SedaDNA reveals regional climate and bedrock nutrients as major drivers of terrestrial plant diversity in northern Fennoscandia.

## INTRODUCTION

Our ability to counter the current loss of biodiversity is dependent on how well we understand the causes of its change through time. However, the trajectory of biodiversity, especially in response to ongoing climate change, is vigorously debated (*1*, *2*), with discrepancy among short-term biodiversity patterns at global, regional, and local scales, whereby local processes may compensate or even counteract global trends (*3*). Our understanding of how a species pool, defined as “a set of species occurring in a particular region that can potentially inhabit a site because of suitable local ecological conditions” (*4*), affects biodiversity patterns through time has so far been restricted by a lack of temporal depth in contemporary ecology and limited resolution in paleoecology. However, ancient DNA can provide refined insight into temporal trends of plant diversity due to high detectability and improved taxonomic resolution (*5*, *6*).

The largest impact of ongoing climate change is expected to be at high latitudes (*7*). Field and modeling analyses suggest plant species richness will increase at high latitudes in Europe as summer temperature increases (*8*). Short-term observational studies, however, suggest that colonization by terrestrial species is lagging behind shifts in temperature isotherms (*9*), which can be compensated in the short term by local extinction lags (*1*). Therefore, studies addressing richness and regional species pool development at high latitudes and at different spatiotemporal scales are warranted to increase our understanding of biodiversity responses to ongoing climate change.

Changes in species richness by other drivers, such as nutrient levels, species introductions, and dispersal lags, are often context dependent and hence difficult to predict. For example, edaphic variation, including variation in nutrient content, is hypothesized to strongly influence establishment, ecological drift, and niche selection, which all affect the regional species pool, and this, in turn, affects richness as local communities assemble from the regional pool (*10*). Experimental approaches have shown a nonlinear impact of fertilization (the addition of essential nutrients) on Arctic plant richness and their ecosystem functions (*11*). An overall greater species richness has been reported from calcareous as compared with siliceous bedrock areas in the European Alps and northern Fennoscandia, whereby leaching of the former produces neutral to acidic microenvironments, providing a mosaic of habitats that may promote species establishment and increase richness at the local scale (*12*, *13*). Human land use may increase richness on fertile soils (*14*), although global analysis shows richness loss from severely affected habitats (*15*). However, the overall human impact at high latitudes in Europe is low (*16*). There is also evidence that the trajectory of succession, particularly soil formation, after glacier retreat varies because of both abiotic and biotic factors (*17*, *18*). Furthermore, it has been found that regional plant species richness increases with time since the land was deglaciated, suggesting that the legacy of past glaciation is still detectable today, whereas local richness may be determined by habitat factors (*19*).

There is a clear need for long-term data at local and regional scales to better understand biodiversity trends (*20*). Paleoecological studies, especially those based on pollen (palynological) analyses, provide direct long-term evidence of plant biodiversity change and have been widely used to estimate effects of climate changes on richness (*21*–*23*). Contrasting richness patterns have been observed in North West Europe over the postglacial period [~15 thousand calibrated years (ka) before present to present]. In the boreal region (Scotland, Fennoscandia, Iceland, Baltic States, and Northwest Russia), pollen richness shows an initial increase during the early colonization period with a peak around 12 ka, followed by an overall decrease from 11.7 to 7.0 ka due to gradual closure of forest, and an increase to near-peak levels by historical times due to deforestation and agricultural practices (*22*). This latter increase is consistent with a study of sites from Norway over the past 8 ka, which showed a continuous increase in richness that was attributed to the hypothesis of postglacial dispersal limitation (*23*). In the far north of Fennoscandia, however, a study spanning a forest to shrub-tundra gradient shows an inconsistent richness pattern through the Holocene (11.7 ka to recent) (*24*). These studies highlight the challenges of comparing pollen richness across different vegetation zones, which is confounded by inferences based on pollen being affected by overabundance of a few taxa (swamping), underrepresentation of other taxa, or low abundance of all taxa, the latter of which is a particular problem above the treeline (*25*).

An alternative, emerging proxy for examining long-term, regional-scale plant richness and species pool trends is sedimentary ancient DNA (sedaDNA). Compared to pollen, sedaDNA provides higher taxonomic resolution, has fewer problems with swamping, and is considered to only represent the local plant community (*6*, *26*, *27*). In a small catchment, sedaDNA may, therefore, also register the effect of drivers on a local rather than regional scale (*28*). Ground truthing studies show a strong correlation between modern sedimentary DNA–inferred richness and richness of modern vegetation around lake catchments (*29*). However, in contrast to pollen, sedaDNA studies have focused almost exclusively on single sites [but see (*28*, *30*, *31*)], thereby limiting our spatial understanding of richness and regional species pool patterns. A key challenge to combining multisite sedaDNA data from lake cores is that data need to be directly comparable, both within (temporal) and between (spatial) sites; otherwise, biased richness estimates resulting from data quality problems could potentially lead to erroneous local- and regional-scale inferences.

Here, we generate the largest lake sedaDNA dataset to date, consisting of 387 dated samples from 10 sites in northern Fennoscandia, using vascular plant metabarcoding. We harmonize the entire dataset using standardized taxonomy and novel data quality control (QC) measures that will be highly applicable to the wider environmental DNA community. Capitalizing on this harmonized high-resolution taxonomic dataset, we estimate taxonomic richness and the regional species pool throughout the Holocene. We compare richness to two potential drivers—climate and local nutrient conditions. We find that temperature and soil nutrients are important drivers but suggest that three other untested drivers—dispersal lags, soil development, and habitat diversification—may also provide an important mechanism for plant richness changes through time. By providing refined paleoecological insights, sedaDNA data are well positioned to increase the precision of integrative ecological models for predicting the consequences of ongoing climate change.

## RESULTS

### Age-depth models

**Fig. 1:**
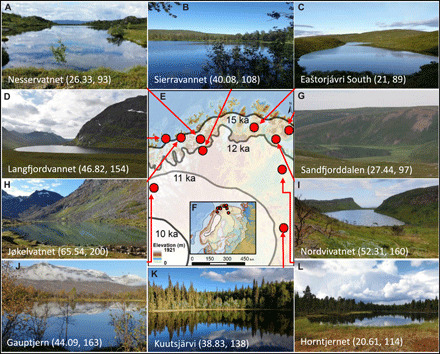
Geographical setting of the study area and images of the 10 lakes. The extent of the Scandinavian ice sheet [the most credible extent of (*79*)] at 21.0 (inset only), 15.0, 12.0, 11.0 (not in inset), and 10.0 ka is indicated by semitransparent layers. Purple polygons in the inset indicate present-day coastline. Lake names are followed by mean taxonomic and accumulated richness recorded in each lake. See dataset S1 for further site information. Map data source: European Environment Agency; photo credits: Jøkelvatnet, L. Topstad; Sandfjorddalen, L. E. Støvern; Langfjordvannet and Eaštorjávri South, Dilli P. Rijal; Kuutsjärvi, Karin Helmens; all others, Inger G. Alsos.

**Fig. 2:**
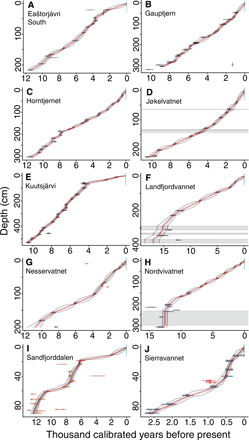
Bayesian age-depth models for 10 lakes from northern Fennoscandia. Colors for calibrated radiocarbon dates follow fig. S1. Excluded dates are in red, and inferred slumps are shown with gray shading. Median modeled ages are indicated by the red lines, with the bubbles representing 95% credibility intervals.

We constructed Bayesian age-depth models for 10 lake sites (Figs. 1 and 2 and fig. S1) to estimate the age of each sedaDNA sample. Because the cores were all central or near-central lake locations and the lakes were medium to small (0.8 to 55.3 ha; dataset S1) with, in most cases, only one depositional basin, the age-depth curves were approximately linear or curvilinear with three exceptions (Fig. 2 and fig. S1). Kuutsjärvi had a distinct reduction in sedimentation rate from around 4.0 ka (Fig. 2E). Lake Sandfjorddalen had stepped changes in the sedimentation rate with possible hiatuses in the Early Holocene (11.0 to 8.0 ka) and Late Holocene between 6.0 and 2.0 ka (Fig. 2I). This probably reflects its position in the valley floor as a flow-through lake (Fig. 1G). Last, Sierravannet had a distinct upturn in the accumulation rate around 0.6 ka to present, which occurs after a putative flood event at ca. 48- to 40-cm composite depth (equivalent to ca. 0.6 ka; Fig. 2J). This 8-cm layer is characterized by a dark band in the visible stratigraphy, a rapid decrease and then increase in organic carbon [loss on ignition (LOI)], and two older-than-expected dates, which were consequently not included in the age-depth model. Given that this lake has the largest catchment area and there is a change in lithology, this is probably the result of flooding from the upstream lakes and fluvial network. The terrestrial plant taxonomic richness trends are unaffected by the removal of the four sedaDNA samples that fall within this flood event window (fig. S2). For the interpretation of the sedaDNA records, the age-depth models provided similar temporal resolution of 158 to 616 years per sample for all lakes except Sierravannet, which had 63 years per sample. Six of the sedimentary records covered the entire Holocene (Fig. 2 and fig. S1), and all except one (Sierravannet) covered the three periods of the Holocene (Early, 11.7 to 8.3 ka; Middle, 8.3 to 4.25 ka; and Late Holocene, 4.25 to 0.0 ka), although the Nesservatnet record was reduced to the Late Holocene after removal of low-quality sedaDNA samples (see below). For two records that extend into the Late Pleistocene (Langfjordvannet and Nordvivatnet), the age-depth models are not well constrained in the Late Glacial period (Fig. 2, F and H). For Langfjordvannet, three hiatuses were inferred by Otterå (*32*). We tentatively infer a single hiatus for Nordvivatnet (Fig. 2H), which could have been caused by a rock slide and is based on the recovery of glacially scarred stones (impressions shown in visible stratigraphy in fig. S1H), low organic carbon (LOI), and comparable radiocarbon dates.

### sedaDNA data quality assessment

**Fig. 3:**
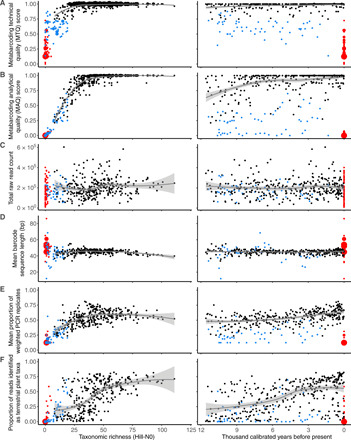
Correlations between taxonomic richness (left) and time (right) against six measures of sedaDNA data quality. Sample age minimally affects MTQ (**A**) and MAQ (**B**) scores, total raw read count (**C**), mean barcode sequence length (**D**), and the mean proportion of weighted PCR replicates (**E**) across the entire dataset, although the proportion of reads identified as terrestrial plant (**F**) increases through time. Data in black, samples that passed QC; blue, samples that failed QC; red, negative controls. Fitted Loess-smoothed lines along with one SE envelope are for samples that passed QC. Data for individual lakes are presented in fig. S4.

Across our 10 lake sediment records, we generated 91.6 million raw sequence reads from 387 sediment samples and 90 control samples. We observed notable differences in data quality between samples both within and between lake records during preliminary data exploration. We therefore sought to develop QC criteria to allow for the objective removal of poor-quality samples, which may have been affected by methodological issues (such as extract inhibition) or ultralow template concentration. We consider samples to be of poor quality if the most read-abundant amplicon sequences, which likely reflect the most abundant template sequences, could only be sporadically replicated across our eight polymerase chain reaction (PCR) replicates as sometimes occur for the negative controls, for example. We therefore developed two statistics based on the proportion of positive PCR detections across the 10 most read-abundant amplicon sequences within a sample (out of a possible total of 80 detections). We first developed a metabarcoding technical quality (MTQ) score to assess metabarcoding success on a per-sample basis, which is based on the 10 most read-abundant sequences before any taxonomic identification. We next developed a metabarcoding analytical quality (MAQ) score to assess the success of recovering sequences of interest. This score is the same as the MTQ score, except that the 10 most read-abundant and taxonomically identified sequences, after removing potential false positives (see Materials and Methods and dataset S6), were used. Sources of MTQ and MAQ score divergence include the coamplification of unidentified and/or contaminant sequences. For all samples across the dataset, we examined the distribution of MTQ and MAQ scores and used these to infer that samples should require an MTQ score of ≥0.75 (at least 60 detections) and MAQ score of ≥0.2 (at least 16 detections) to pass QC and be included in downstream analyses (Fig. 3 and figs. S3 and S4). This cutoff excluded all negative controls, which had an MTQ score of <0.75 and an MAQ score of <0.1. For 16 samples that were extracted more than once, we used data from the extraction replicate with the highest MAQ score (dataset S4).

After applying our QC thresholds, we retained 316 samples (Fig. 3), with 12 to 55 samples retained per record (fig. S3, table S1, and dataset S4). We retained 402 barcode sequences, which were collapsed to 346 taxa (datasets S6 and S7). Of these, 50% could be assigned to the species level (datasets S6 and S7). As our focus was on terrestrial plant diversity, we excluded 13 algae and 36 aquatic plant taxa (dataset S6). Nine taxa were only present in samples that failed QC. Thus, our final dataset retained 288 terrestrial plant taxa detected in 316 samples, with between 89 and 200 taxa recorded from each lake record (Fig. 1). We note that all 288 taxa are known from the northern Fennoscandia today and are therefore a part of the known regional species pool. We next explored the relationship between taxonomic richness and/or sample age against *MTQ/MAQ* scores and four other measures of sedaDNA quality, with each measure calculated on a per-sample basis.

#### MTQ and MAQ scores

Both scores correlate with richness when richness is low (<25 to 30; Fig. 3, A and B), which is likely an artifact of the requirement that the 10 best represented barcode sequences are required to calculate these scores. At higher richness values, both MTQ and MAQ scores are uniformly high. MTQ scores are minimally affected by sample age (Fig. 3A and fig. S4A), although samples older than ~8.0 ka tend to have lower MAQ scores (Fig. 3B), which is driven by the Eaštorjávri South, Langfjordvannet, Kuutsjärvi, Nesservatnet, and, to a lesser extent, Jøkelvatnet records (fig. S4B).

#### Raw read count summed across PCR replicates

Richness may be influenced by differences in total sequencing depth between samples, whereby we would expect increased total depth to correlate with richness as the likelihood of detecting read-rare taxa is increased [e.g., (*33*)]. However, we do not observe a relationship between richness and raw read count (Fig. 3C), suggesting that differences in sequencing depth (range, 9567 to 604,068 reads per sample; dataset S4) do not influence richness in our dataset. This is consistent with the results of the rarefied richness analyses (dataset S8). We also do not observe a relationship between sample age and sequencing depth (Fig. 3 and fig. S4C), suggesting that there is no temporal or stratigraphic bias in our ability to generate raw reads. We note that samples that passed or failed QC had comparable total sequencing depths.

#### Mean length of identified barcode sequences through time

As ancient DNA fragments into shorter molecules over time (*34*), a reduction in mean barcode sequence length with sample age may suggest that longer barcode sequences are no longer preserved thus biasing estimates of temporal richness patterns. However, this assumes that barcode sequence length is independent of taxonomic group and/or ecological community composition, which may not always be the case. We do not observe a decrease in mean barcode sequence length with age in samples that pass QC (Fig. 3D and fig. S4D). Samples that fail QC are often mean length outliers that show no relationship to age but are rather an artifact of small sample sizes. Minor decreases in mean barcode sequence length with sample age are observed for Kuutsjärvi and Langfjordvannet (fig. S4D).

#### Mean proportion of weighted PCR replicates

The mean proportion of weighted PCR replicates (wtRep) provide a proxy for mean taxon detectability in samples. If barcode sequence template concentrations in sedaDNA extracts are low, then we would expect recovered richness to be limited, because of dropout, with a corresponding reduction in detectability of taxa that are observed. Consistent with this expectation, we find that mean wtRep values are comparable for samples with richness >25 to 30 (Fig. 3E), but that mean wtRep and richness are correlated when richness is <25 to 30. However, we observe only a modest decrease in mean wtRep values with sample age (Fig. 3E), suggesting that taxon detectability is not affected by age. The greatest age-related reductions in mean wtRep values are in samples from Langfjordvannet, Kuutsjärvi, Nesservatnet, and, to a lesser extent, Jøkelvatnet (fig. S4E). We note, a posteriori, that mean wtRep and MAQ scores may not be independent measures, especially for samples with low richness (<25 to 30).

#### The proportion of raw reads assigned to terrestrial plant taxa

If there is coamplification of nonterrestrial plant, algae, contaminant, and/or other off-target molecules, then terrestrial plant richness may be reduced by swamping. We observe that at least ~40% of reads are required to be identified as terrestrial plant taxa for richness to exceed ~60 taxa (Fig. 3F). When richness is <60 taxa, we observe that the proportion of reads identified as terrestrial plant taxa decreases with richness (Fig. 3F). However, we note that there is large variance around this trend and so, for example, samples from the Middle Holocene of Sandfjorddalen have comparable richness to Early Holocene samples from Gauptjern (fig. S3), although reads from the former are swamped by reads from aquatic plant taxa (fig. S5). Across the entire dataset, there is a gradual reduction in the proportion of reads identified as terrestrial plant taxa with sample age, which is driven by the Eaštorjávri South, Kuutsjärvi, and Nesservatnet records (Fig. 3 and figs. S4F and S5). Samples that failed QC tended to have a lower proportion of reads identified as terrestrial plant taxa (Fig. 3F and figs. S4F and S5).

Overall, we found that the MTQ and MAQ score QC thresholds removed the worst-performing samples. Our data quality assessment suggests that the records with the best sedaDNA quality are Gauptjern, Horntjernet, Nordvivatnet, Sandfjorddalen, and Sierravannet, whereas Early Holocene samples from Eaštorjávri South, Kuutsjärvi, Langfjordvannet, and Jøkelvatnet were of lower quality (figs. S4 and S5).

### Taxonomic and accumulated richness

**Fig. 4:**
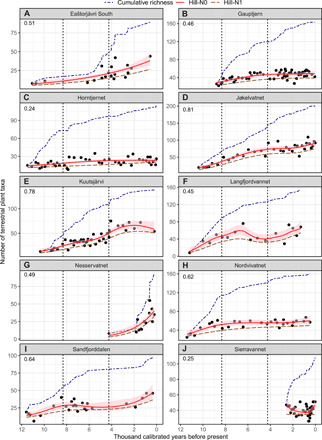
Holocene patterns of local terrestrial plant richness in 10 lake catchments from northern Fennoscandia. The fitted lines for taxonomic richness (Hill-N0; solid red line) and Hill-N1 (dashed brown line) are based on generalized additive models (GAMs) with a time-first autoregressive process (GAM-CAR1). The 95% confidence intervals of Hill-N0 are in pink shading. Numbers in the top-left corner of each plot represent adjusted *R*^2^ values for the GAM-CAR1 models. The cumulative count of taxa (accumulated richness) through time is given as a blue dot-dashed line. The Early (11.7 to 8.3 ka), Middle (8.3 to 4.25 ka), and Late Holocene (4.25 to 0.0 ka) periods are indicated by vertical dotted lines. Note the difference in scale on the *y* axes.

To evaluate how richness changed through time, we used the count of taxa observed in each sample (taxonomic richness, Hill-N0) and the accumulated count of taxa across samples within a lake (accumulated richness). At the local scale, within a lake catchment, the highest accumulated richness was recorded at Jøkelvatnet (200 taxa; Fig. 4D), which today drains a catchment that has a Late Holocene glacier in its upper reaches (Fig. 1H and section S1). Accumulated richness was also high at Gauptjern, which is at the border between pine and birch forest (Figs. 1J and 4B), and at Nordvivatnet and Langfjordvannet (Fig. 4, F and H), which have a mixture of heathland, birch forest, and scree slope in their catchments (Fig. 1, D and I). Somewhat lower accumulated richness values were calculated at the two sites in pine forest, Horntjernet (Figs. 1L and 4C) and Kuutsjärvi (Figs. 1B and 4E), and at Sierravannet, a site with birch forest and pine and larch plantations (Figs. 1K and 4J). The two shrub-tundra sites, Eaštorjávri South (Figs. 1C and 4A) and Sandfjorddalen (Figs. 1G and 4I), had smaller accumulated richness, similar to Nesservatnet, which is surrounded by heathland/mires (93 taxa) and located on the small island of Årøya (Figs. 1A and 4G).

There were clear differences among lakes, both in the overall mean taxonomic richness and in the change in taxonomic richness over the Holocene (Fig. 4). The mean (± SD) taxonomic richness ranged from 20.6 (± 6.4) at Horntjernet to 65.5 (± 24.5) at Jøkelvatnet, whereas Hill-N1 ranged from 14.9 (± 7.8) at Eaštorjávri South to 52.4 (± 20.5) at Jøkelvatnet (Fig. 4 and table S1). The rarefied richness based on read counts per sample showed a strong correlation with taxonomic richness (*R* = 0.82 to 0.99; dataset S8), suggesting that the observed pattern was not affected by sequencing depth. The Hill-N1 (common taxa) showed temporal patterns that mirrored those of taxonomic richness for all the lakes except Sierravannet (Fig. 4).

We observed a significant effect of the age of samples on taxonomic richness as indicated by statistically significant smooth terms in generalized additive models (GAMs) (table S2), except for Sierravannet, which only covered 2.6 ka, and where diversity suddenly dropped around 0.6 ka, corresponding to a putative flood event (fig. S2). For two of the lakes, Eaštorjávri South and Nesservatnet, a near linear pattern of increase in taxonomic richness through time [estimated degree of freedom (edf) = 1] was recovered. On the other hand, Langfjordvannet had the most complex pattern of increase in richness (edf = 5.93; table S2). The steepest increase was seen in the Early and Middle Holocene for most lakes. Only at three sites, Nordvivatnet, Horntjernet, and Gauptjern, did richness reach a plateau during the Late Holocene; for most lakes, no leveling off was observed suggesting that richness is still increasing (Fig. 4).

**Fig. 5:**
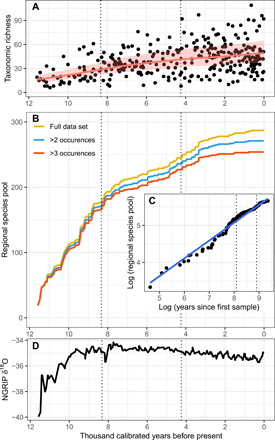
The regional pattern of taxonomic richness, regional species pool, and accumulation rate, and North Greenland Ice Core Project temperature proxy across the Holocene of northern Fennoscandia. (**A**) Taxonomic richness: Count of taxa detected per sample (*n* = 316) along with the 95% confidence interval (pink shading) of the fitted line (solid red line) based on a GAMM-CAR1 [SD of random error = 0.38, estimated autocorrelation (ϕ) = 0.23], (**B**) the regional species pool (defined as the cumulative count of taxa from the region) for the full dataset as well as excluding rare taxa (defined as one or two occurrences across the entire dataset), (**C**) the rate of regional species pool growth (dotted vertical lines represent Holocene periods as in other panels), and (**D**) variation in temperature reflected by North Greenland Ice Core Project (NGRIP) δ^18^O values (*74*), with temperature being positively correlated with δ^18^O values. The overall patterns remain the same if two shorter cores spanning only the Late Holocene are excluded or higher data quality thresholds are used (fig. S6). The Early (12.0 to 8.3 ka), Middle (8.3 to 4.25 ka), and Late Holocene (4.25 to 0.0 ka) periods are indicated by dotted vertical lines.

We evaluated the temporal trend of taxonomic richness at the regional scale, using data from all 316 retained samples and a generalized additive mixed model (GAMM). Taxonomic richness (fitted ± SE) showed a continuous increase from the Early to Middle Holocene (16.3 ± 1.2 to 41.2 ± 1.1 taxa, with 28.8 ± 1.1 at the Early/Middle Holocene transition; Fig. 5A), whereas only a minor increase was observed during the Late Holocene (41.3 ± 1.1 to 49.2 ± 1.2 taxa).

### Regional species pool

The regional species pool, defined as the accumulated count of taxa from all 316 retained samples across the Holocene, showed a strong increase during the Early Holocene (Fig. 5, B and C, and fig. S6). The regional species pool increased monotonically from the Early Holocene to ca. 7.0 ka (42.37 taxa per millenium), and the rate slowed in the period ca. 7.0 to 5.0 ka (9.35 taxa per millenium) followed by an uptick from 5.0 to 3.3 ka (21.82 taxa per millenium) before stabilizing. The regional species pool leveled off over the past three millennia with an increase of just 16 taxa (5.6% of the total). The overall trajectory of the regional species pool is unaffected by (i) the exclusion of taxa with one or two occurrences in the dataset (Fig. 5B), (ii) using more stringent MAQ score thresholds for sample inclusion (up to MAQ ≥ 0.6; fig. S6A), or (iii) the exclusion of two short records spanning only the Late Holocene (fig. S6B).

**Fig. 6:**
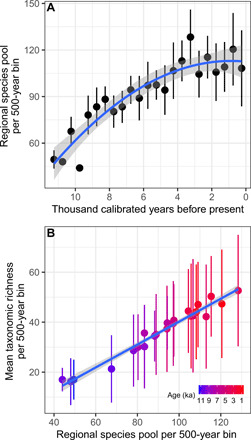
Relationship between regional species pool in 500-year time bins, time, and mean taxonomic richness. Correlation between (**A**) the regional species pool (total number of taxa observed in five samples per 500-year time bin) against bin age with a second-order polynomial fit, and (**B**) the regional species pool (as defined above) and mean taxonomic richness of terrestrial plants (*N*: 5 to 34 samples per bin) in 500-year bins in northern Fennoscandia.

We next estimated the regional species pool within 500-year time bins using cumulative taxonomic richness within bins while controlling for uneven sample size between bins. We found that this estimated regional species pool increased with time during the Early and Middle Holocene, before reaching a plateau after ~3 ka (Fig. 6A). The pattern from this alternative approach to estimating the regional species pool, which controls for periods of extinction within the Holocene, is broadly consistent with that derived from the accumulated count of detected taxa across the whole Holocene. We found a strong positive correlation between mean taxonomic richness across catchments and estimated regional species pool size per 500-year bin, with 86% of the variation in mean taxonomic richness explained by the regional species pool size (*R*^2^_adj_ = 0.86, *F*_2,20_ = 66.07, *P* < 0.0001; Fig. 6B).

### Impact of regional climate and local nutrient availability on plant richness

**Fig. 7:**
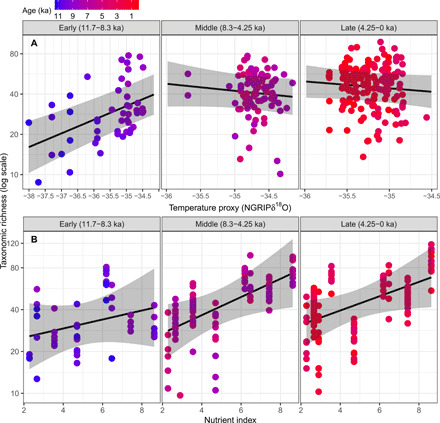
Impact of climate and nutrient index on taxonomic richness. Linear mixed effects model showing (**A**) the impact of regional climate and (**B**) nutrient index on taxonomic richness of terrestrial plants for three periods of the Holocene. Temperature is positively correlated with δ^18^O values. Two samples with NGRIP δ^18^O lower than −39 were not included in the analysis. Note difference in scale on the *x* axes. Taxonomic richness is log-transformed, but respective nontransformed values are displayed on the *y* axes.

We used (i) oxygen isotope (δ^18^O) values from the North Greenland Ice Core Project (NGRIP) (*35*) as a proxy for temperature to assess the impact of regional climate on taxonomic richness during the three different periods of the Holocene (Early, Middle, and Late) and (ii) a new nutrient index based on rock type and its weatherability to assess how nutrient availability in the catchment area affects richness (dataset S1 and Eq. 2). Sample age was included as a random slope effect to account for different temporal rates of change in taxonomic richness among lakes. In considering the positive association between nutrient index and taxonomic richness of lakes for different periods of the Holocene, we highlight that our nutrient index is based on bedrock weathering, and the potential release of phosphorus, potassium, and calcium, which acts as a surrogate for alkalinity. Fixed effects (temperature, nutrient availability, and their interaction with Holocene periods) explained 50.3% of the variation in taxonomic richness, whereas random effects explained an additional 25%. In the Early and Late Holocene, temperature changed linearly through time at a rate (± SE) of 0.92 (± 0.07) and −0.13 (± 0.01) δ^18^O/ka, respectively, whereas there was no directional change in temperature across the Middle Holocene (Fig. 5D). Temperature had a significantly positive relationship with richness in the Early Holocene (β = 0.24, SE = 0.042, *P* < 0.001) and a slight, but statistically nonsignificant, negative relationship in the Middle (β = −0.12, SE = 0.10, *P* = 0.25) and Late Holocene (β = −0.12, SE = 0.10, *P* = 0.22; Fig. 7A). There was a significant interaction between Holocene period and temperature (*F*_2,296_ = 8.30, *P* = 0.0003), and between Holocene period and nutrient availability (*F*_2,268_ = 5.48, *P* = 0.005). The nutrient-richness relationship was strongest for the Middle Holocene (β = 0.15, SE = 0.06, *P* = 0.04), weaker for the Late Holocene (β = 0.12, SE = 0.06, *P* = 0.07), and weakest for the Early Holocene (β = 0.075, SE = 0.078, *P* = 0.35; Fig. 7B). The effect of nutrient index on taxonomic richness was therefore strongest during the Middle Holocene, when the impact of climate was weaker.

## DISCUSSION

### The ability of sedaDNA to capture plant taxonomic richness

We present local and regional trends in taxonomic richness and the regional species pool of terrestrial plants, as inferred from 10 Holocene sedaDNA records covering a broad environmental gradient in northern Fennoscandia. Our development of new QC metrics to standardize data, together with improved taxonomic precision and known source areas (hydrological catchments of the lakes), allows for meaningful estimates of taxonomic richness, its spatial variation, and temporal trends, which show a unique increasing pattern of terrestrial plant richness over the Holocene. Individual differences were observed among our sites, but our novel combined and standardized sedaDNA analyses of 10 sites provide a superior representation of the overall regional patterns in plant taxonomic richness and regional species pool development over the Holocene as compared to traditional approaches.

The mean taxonomic richness of terrestrial plants per sample and site (~21 to 66) is higher than that recovered for northern boreal sites based on pollen analyses [~20 taxa, (*22*)] but similar to pollen estimates from the Alps and Mediterranean [~30 taxa, (*22*)]. The detected richness values are within the range that has been found in other recent studies of sedaDNA from northern sites [20 to 70 taxa; (*28*, *36*)], although some shrub tundra [~13 taxa, (*37*)] and High Arctic [5 to 30 taxa, (*38*)] sites have notably lower estimates. Nevertheless, our results are consistent with other sedaDNA analyses that detect more taxa than pollen counts (*6*, *26*, *28*). Together with improved geographic fidelity due to a local signal, sedaDNA thereby improves our understanding of the spatial patterns and scale dependency of past plant diversity.

We are aware that our approach has several limitations. First, one should be aware that sedaDNA analyses based on p6-loop metabarcoding has taxonomic biases, as some species-rich families such as Salicaceae, Poaceae, and Cyperaceae are poorly resolved because of haplotype sharing (*31*, *39*). We have used a local reference database to maximize taxonomic resolution and note that this is also an issue for traditional palynological proxies. Second, we acknowledge that the initial steep increase in our regional species pool estimates at the start of the Holocene constitutes a sampling probability artifact, as plant taxa are known from the region in the Late Glacial (*40*, *41*). However, we note that a consistent steep increase continues throughout the Early Holocene interval, which we do not consider to be a sampling artifact. Third, we assume that the NGRIP record is representative of the climate of northern Fennoscandia. This record is in accordance with local climate reconstructions in northernmost Fennoscandia based on macrofossils and pollen, although local variation does exist especially because of the proximity of the Norwegian Coastal Current, which is an extension of the Atlantic Gulf Stream (*40*). Last, we have not considered human impact as a potential driver of the biodiversity trends documented here but emphasize that this is considered to have been low in northern Fennoscandia as compared to other regions in Europe (*16*).

The temporal patterns evaluated here rely on the assumption that our ability to detect plant taxa in sedaDNA is not affected by differential preservation, because of sample age or methodological problems such as DNA extract inhibition. Here, we discarded samples of poor quality that had metrics comparable to negative controls and thus may have been affected by methodological problems, and we broadly examined the quality of the retained samples. Half of our sites showed no evidence of declining sedaDNA quality with sample age, whereas the remainder had reduced quality in the Early Holocene interval. The fact that our samples generally exhibited good sedaDNA quality throughout the study interval is likely due to a combination of excellent DNA preservation in the cold environments of high latitudes (*42*) and the young age of the samples (<11.7 ka) relative to the known upper limit of ancient DNA preservation [~1200 ka, (*43*)]. As multisite sedaDNA studies become common, it will be crucial that data quality is scrutinized and, where possible, standardized to allow for biologically meaningful comparisons between sites.

### A steep Early Holocene increase in plant richness

The highest rate of increase in the regional species pool is observed in the Early Holocene (11.7 to 8.3 ka). Because of their notable correlation, we cannot distinguish the effect of dispersal lags from temperature, and both factors likely contributed to the observed increase in diversity. Climate was also the driver for deglaciation, which increased the area available for colonization. Three of our records span a longer time period than examined here (Langfjordvannet, 16.7 ka; Nordvivatnet, 12.7 ka; Sandfjorddalen, 12.5 ka; fig. S1, F, H, and I), and they, as well as macrofossil [14.5 ka, (*41*)] and pollen records [13.9 ka, (*40*)] from other coastal sites in northern Fennoscandia, show that an Arctic pioneer vegetation established toward the end of the Younger Dryas period (12.9 to 11.7 ka) and into the Early Holocene. Thus, a regional species pool already existed at least along the coast at the start of our study period, which is not the case for some of the inland sites (Gauptjern, Horntjernet, and Kuutsjärvi) that were deglaciated after the onset of the Holocene (Fig. 1). Nevertheless, all sites and the region as a whole exhibit a strong increase in richness independent of location relative to deglaciation.

During the rapid warming at 11.7 to 10 ka, we find an especially high increase in richness and the regional species pool. It is likely that nutrient release started immediately after deglaciation when liquid water was abundant (*44*) and light-demanding and disturbance-tolerant pioneer species could have survived on the young microhabitats with poorly developed soils and thus showed weak overall association with the nutrient index. Additional factors other than climate and the availability of nutrients and land may have influenced richness in this period. For example, biotic factors such as low levels of competition may have facilitated establishment (*45*), and abiotic factors, particularly paraglacial processes, may have produced disturbance at the local scale (*46*). On the other hand, dispersal lags may have limited taxonomic richness and the regional species pool, as for example, the 400-year time lag between climate and arrival of birch woodland that was estimated on the basis of plant macrofossils recovered from sediment cores (*47*). Nevertheless, the overall rapid increase in diversity in an early phase of colonization is also recorded in pollen studies (*22*) and expected given that they cover the development from pioneer to more established vegetation communities. Our richness patterns show a continued strong increase after ~11 ka, when the major expansion of birch forest took place, and after ~10 ka when pine expanded into the region (*24*). Thus, in contrast to the decrease in richness due to forest expansion observed in pollen studies (*14*, *22*), we found a general increase in richness throughout the Early Holocene. This may be because sedaDNA analyses are less sensitive to swamping by trees than pollen analyses and therefore better reflect habitat complexity (*26*–*28*).

### Middle Holocene plant richness continued to increase

The continued increase in richness, at the local and regional scales, and the reduced rate of increase in the regional species pool during the Middle Holocene (8.3 to 4.2 ka) were not related to climate. The NGRIP record shows a temperature peak (end of the Holocene Thermal Maximum) followed by slight cooling during this period. Richness leveled off in only two lakes (Nordvivatnet and Sandfjorddalen) and one lake (Langfjordvannet) showed a hump in richness, which we assume is due to local factors. For Gauptjern, palynological richness fluctuates around eight taxa for this period (*48*), whereas our sedaDNA data show a clear increase. Pollen studies from northern Fennoscandia have shown contrasting patterns through this period, including stable levels of richness along a spruce-pine-birch tundra transect (*24*) and an overall increase in richness (*23*). The closest sites previously studied for sedaDNA show stable richness at Varanger in northeasternmost Norway (*37*), increasing richness in Svalbard (*38*), and fluctuating high richness in the Polar Urals (*36*). Seen from a regional perspective, our richness curves are similar to those found in the temperate zone of Europe, where a Middle Holocene richness increase is inferred to be due to human impact but differ from those of the boreal zone (*22*), probably because of a lower influence of Holocene tree expansion in the sedaDNA data. Thus, in contrast to many pollen studies, our sedaDNA data show an increase in taxonomic richness during the Middle Holocene.

As the climate was relatively warm and stable during this period, we suggest that bedrock leaching and nutrient release would have increased and contributed to the development of soils (*49*), thereby promoting richness, especially on nutrient-rich bedrocks, and growth of the regional species pool. Relevant here is the fine spatial scale of the calcareous (alkaline) and siliceous (acidic) bedrock in northern Fennoscandia, with small and often linear outcrops of metamorphic carbonate (*13*). This contrasts with the large calcareous limestone massifs found in younger geologies such as the European Alps, which have been shown to have positive effects on diversity over both short and long time scales (*12*). We speculate that continued increases in richness and the regional species pool may also have been mostly driven by dispersal lags, resulting in delayed establishment of some taxa in the region, and habitat diversification allowing for a greater variety of niches, including the development of heathland, meadows, and mires (*16*).

### Late Holocene plant richness neared a plateau

The rate of increase in taxonomic richness at the regional scale slowed during the Late Holocene, and the regional species pool clearly leveled off during the past three millennia, suggesting that an equilibrium level may have been reached. The slight cooling and well-known instability in this period (*50*) did not substantially affect richness. Palynological richness in northern Fennoscandia increases slightly (*48*) or is variable (*24*) during this period. Richness also increases at sites in the boreal and temperate regions of Europe, mainly because of human land use impacts (*22*, *51*). The reason for leveling off at the regional scale in northern Fennoscandia is likely due to equilibrium of the regional species pool and the overall low impact of human land use within the catchments.

In contrast to the regional scale, our data suggest that the taxonomic richness in lake catchments is still increasing. This is in contrast to what has been observed in studies of modern vegetation, where there appears to be no effect of time since glaciation for local (plot level) richness, whereas a legacy of the ice age is inferred for richness at the pan-Arctic (floristic region) scale (*19*). This apparent contradiction may be the result of scale and environmental spatial variation. Our catchments are larger than the plots studied by Stewart *et al*. (*19*) and may therefore allow for coexistence of different vegetation types. Ongoing warm and possibly wet conditions would have continued to release bedrock nutrients and allow for further soil development, thereby promoting habitat diversification in the Late Holocene. For instance, soils developing slowly on hard felsic and mafic rocks have low buffering capacity, which results in nutrient loss and the partial development of oligotrophic vegetation types such as acid heaths and ombrotrophic mires. These have their own floras, and some species are restricted to these environments. Mires and heath vegetation expanded in the region during the Late Holocene (*16*, *24*). Depending on the local bedrock, a given area may thus gradually come to include additional vegetation types, allowing more hardy species to grow and total richness to increase while retaining the more demanding species in more favorable areas of the catchment. In addition, infilling of the lake creates wetland zones that also may include terrestrial taxa. Thus, a continued increase in taxonomic richness at the local scale may be due to habitat diversification.

### The future trajectory of plant richness

We suggest that plant diversity will depart from equilibrium and increase, perhaps markedly, in northern Fennoscandia as a consequence of the northward movement of warm adapted species due to ongoing climate warming [sensu (*1*)]. Expanding human impacts within the region may further affect richness. However, we speculate that increases in diversity may be tempered by dispersal lags and the time taken for habitats to diversify as is hinted at by our Holocene dataset. We further speculate that losses of cold-adapted taxa from the region from ongoing climate warming could be minimal, because of the mountainous topography of northern Fennoscandia providing newly available suitable habitat (*52*). However, it is also possible that cold-adapted species may ultimately extirpate because of competition for the suitable habitats in the long term [sensu (*1*)].

Integration of long-term paleo- and contemporary ecological data will be key to understanding, predicting, and managing the consequences of ongoing climate warming in northern ecosystems. Our study showcases how regional scale sedaDNA data can provide refined paleoecological insights into richness and regional species pools, as compared to traditional proxies, which will increase the precision of integrative ecological models.

## MATERIALS AND METHODS

### Study area, site selection and properties, and fieldwork

The study area covers northernmost Fennoscandia above the Arctic Circle (at 67.75°N to 70.43°N and 19.62°E to 30.02°E) with nine lakes in Norway and one in Finland (Fig. 1 and dataset S1). We selected these 10 sites on the basis of environmental and climatic variables, geographic spread, and vegetation types (section S1). We retrieved sediment cores at seven sites and used previously collected cores for the remaining three. Coring was conducted using a modified Nesje piston corer (*53*) or a rod-operated Multisampler. All cores were kept cold during transport to and storage at dedicated facilities (section S2).

### Core sampling, photography, and LOI analysis

We split piston core sections longitudinally and sampled one half for sedaDNA and LOI analysis in a dedicated clean room facility at The Arctic University Museum of Norway in Tromsø (TMU) (section S3). We took sampling negative controls to monitor for contamination. The remaining half core was retained for high-resolution photography. We extruded and sampled Multisampler cores in the same dedicated clean room. For three previously collected cores, we took sedaDNA samples at the University of Bergen [Langfjordvannet (*32*) and Jøkelvatnet (*54*)] or at Stockholm University [Kuutsjärvi (*55*)], taking sampling negative controls as above. We performed high-resolution imagery at The Arctic University of Norway. We calculated dry mass LOI by igniting the sample at 550°C (*56*) (section S4).

### Composite core construction and age-depth modeling

We opportunistically collected macrofossils for radiocarbon (^14^C) dating during sampling for LOI and sedaDNA, where possible. If additional macrofossils were required, we sieved sediment to concentrate macroscopic plant remains suitable for dating. Two sieves of 500 and 250 μm were stacked while sieving to catch plant macrofossil remains. Ultrapure water from the Milli-Q system was used for sieving and cleaning, and samples were kept cool in Eppendorf tubes with water before shipping for dating. We photographed and identified all macrofossils before their destruction during radiocarbon dating. Samples were radiocarbon dated using accelerator mass spectrometry at the Poznań Radiocarbon Laboratory of the Adam Mickiewicz University, Poland (*57*) (dataset S3).

For multiple core records from the same site, we aligned cores on the basis of combinations of LOI values, visible stratigraphy, and/or radiocarbon dates to create composite core records (fig. S1 and datasets S2 and S3). For Gauptjern, we used the LOI profile and radiocarbon dates produced by Jensen and Vorren (*48*) to guide composite age-depth model construction (fig. S1B and dataset S3). All reported depths are based on the composite cores and begin at the water-sediment interface, which was determined either by its successful capture, field notes, or previously published information (dataset S2). We note that the composite depths reported here differ for two of three previously published records (*32*, *48*, *54*) (dataset S3). For Langfjordvannet, we increased depths by 26 cm to account for the amount of sediment reported missing from the top (*32*), whereas for Gauptjern, we removed 340 cm (water depth) and adjusted remaining depths for differing deposition rates among all cores due to an uneven bedrock surface (*48*).

We constructed Bayesian age-depth models using Bacon v2.3.4 (*58*) in R v3.4.4 (*59*), using the IntCal13 calibration curve (*60*). Each basal modeled age was ≤2 cm below the basal radiocarbon date, with the exception of Langfjordvannet where the basal radiocarbon date falls within a slump that extends to the base of the core. We were unable to confidently model the basal 31 cm of Jøkelvatnet and 22 cm of Kuutsjärvi, as extrapolated ages were highly influenced by accumulation rate priors. We fixed the top of each record to zero, based on the composite cores beginning at the water-sediment interface. The default section thickness of 5 cm was used for all age-depth models, with the exception of Sandfjorddalen, which shrank from the 149 cm collected to 92 cm analyzed. We therefore selected a 2-cm thickness for the Sandfjorddalen age-depth model. We excluded two dates (Poz-108675 and Poz-108983) from the Sierravannet age-depth model that occurred in a putative flood layer (fig. S1J).

### sedaDNA data generation

We performed all pre-PCR steps at the dedicated ancient DNA facilities at TMU, which are in a separate building to post-PCR facilities. We homogenized DNA samples by holding samples on a pulse vortexer for ~1 min. We extracted DNA from 0.25 to 0.35 g of sediment (dataset S4) using a modified form of the Qiagen DNeasy PowerSoil PowerLyzer (Qiagen Norge, Oslo, Norway) protocol, following the protocol of Zimmermann *et al*. (*61*), as modified by Alsos *et al*. (*62*). We included one negative extraction control, consisting of no input, for every 10 sediment extractions. We also re-extracted DNA from 16 samples using modified versions of our protocol (dataset S4). This included six minerogenic samples from Gauptjern, for which we used a carbonate-optimized protocol (*5*). Ten samples from Eaštorjávri South (*n* = 3) or Nesservatnet (*n* = 7) were re-extracted using a protocol that concentrates supernatant after the addition of Solution C3 using a 10K–molecular weight cutoff Vivaspin filter.

We amplified DNA and control extracts using “gh” primers (*63*) that target the vascular plant trnL p6-loop locus of the chloroplast genome (dataset S5). The gh primers were uniquely dual-tagged with an 8- or 9-bp (base pair) tag, modified from Taberlet *et al.* (*64*). We used differing tag lengths to ensure that nucleotide complexity was maintained during amplicon sequencing runs. A total of eight PCR replicates were amplified per extract following Voldstad *et al.* (*38*). We included negative PCR controls, consisting of water as input, to monitor for contamination during the PCR. We additionally included negative and positive PCR controls in the post-PCR laboratory, the latter of which consisted of one of six synthetic sequences (available at https://github.com/pheintzman/metabarcoding) (see also section S5). These post-PCR laboratory controls were added to wells without disturbing other sealed sample and control wells and were used to monitor PCR success. However, they are not comparable to other negative controls and samples, because of exposure to the post-PCR laboratory atmosphere, and so they were excluded from further analysis. We checked for successful amplification using gel electrophoresis (2% agarose).

We pooled up to 384 PCR products (the maximum number of available tags) and then cleaned the resulting pool following Voldstad *et al*. (*38*). Each amplicon pool was then converted into a DNA library at either Tromsø or Fasteris, SA (Switzerland). The Tromsø protocol used the Illumina TruSeq DNA PCR-Free protocol (Illumina Inc., CA, USA) with unique dual indexes, except that the magnetic bead cleanup steps were modified to retain short amplicons, whereas Fasteris used the PCR-free MetaFast protocol to produce single-indexed libraries following Clarke *et al*. (*37*). Each library was sequenced on ~10% of 2× 150-cycle mid-output flow cells on the Illumina NextSeq platform at either Fasteris or the Genomics Support Centre Tromsø (GSCT) at The Arctic University of Norway in Tromsø, or on 50% of a 2× 150-cycle flow cell on the Illumina MiSeq platform at Fasteris. Full sample preparation metadata is provided in dataset S5.

### Bioinformatics

We followed a bioinformatics pipeline that uses a combination of the ObiTools software package (*65*) and custom R scripts (available at https://github.com/Y-Lammers/MergeAndFilter). Briefly, we merged and adapter-trimmed the paired-end reads with SeqPrep (https://github.com/jstjohn/SeqPrep/releases, v1.2). We then demultiplexed the merged data using an 8-bp tag PCR replicate lookup identifier (provided in dataset S5), which ignored the terminal base for 9-bp tags and collapsed identical sequences. We removed putative artifactual sequences from our data, which may have derived from library index swaps or PCR/sequencing errors. For each PCR replicate, we removed sequences represented by ≤2 reads. We next identified sequences that had 100% identity agreement with a local taxonomic reference database (ArctBorBryo) containing 2445 sequences of 815 arctic and 835 boreal vascular plants, as well as 455 bryophytes (*31*, *39*, *66*). In addition, we matched our dataset to the European Molecular Biology Laboratory (rl133) nucleotide reference database. We separately compared our dataset against the barcode sequence of the DNA tracer, with the closest match consisting of 85% identity (see section S6). We therefore consider the DNA tracer not to be present in our dataset. We further removed identified sequences that 100% matched against two lists of potential false positives (https://github.com/Y-Lammers/Metabarcoding_Blacklists) consisting of either synthetic sequences (*n* = 6), or assumed false-positive sequences that represented homopolymer variants of a more read-dominant sequence, a potential random match, or food contaminants (*n* = 111) (dataset S6). We further removed sequences represented by fewer than 10 reads and/or three PCR replicates within the entire dataset, as well as 61 low-frequency sequences that were only retained by analysis of the entire dataset but removed if analyses were conducted on a per-lake basis (dataset S6). If multiple barcode sequences were assigned to the same taxon and co-occurred within samples, then they were assumed to belong to the same taxon and therefore merged using the sum of all assigned reads and the maximum number of PCR replicates (dataset S7). Barcode sequences that were assigned to the same taxon but did not co-occur in samples were kept as separate taxa and labeled with sequential counts (e.g., Asteraceae1 and Asteraceae2). The final taxonomic assignment of the retained sequences was determined using regional botanical taxonomic expertise by Alsos and following the taxonomy of the Panarctic Flora (*67*) and Lid’s Norsk Flora (*68*). We identified two species of *Vaccinium* on the basis of a poly-A region at the 3′ end of the p6-loop locus. If there were ≤5 or >8 As, then barcode sequences were respectively assigned to *V. myrtillus* or *V. vitis-idaea*. We further excluded barcode sequences that were identified above the family level on the basis of alignment to the local reference database ArctBorBryo. Among the identified plant taxa, all aquatic plants (including wetland plants and algae; dataset S6) were excluded and only terrestrial vascular plants and bryophytes were retained (dataset S6). We only included Holocene-aged (11.7 ka to present) samples for downstream analysis. We note that we use the terms taxonomic richness to include taxa identified to various ranks from the species to family levels; sequences for sequence data before taxonomic assignment and filtering, as these may consist of artifacts; and barcode sequences for sequences retained after taxonomic assignment and filtering to remove potential errors.

### Assessment of sedaDNA data quality

Our MTQ and MAQ score thresholds excluded all negative controls, which had an MTQ of <0.75 and an MAQ of <0.1. Across our entire dataset, 16 samples were extracted more than once. We included data from the DNA extract that yielded the greatest MAQ score. In three cases with equal MAQ scores, we selected replicate 1 for inclusion (dataset S4).

After data filtering, we found that there was often large variation in the counts of retained reads between PCR replicates within a sample (from hundreds to hundreds of thousands; dataset S4). Although read-dominant barcode sequences are likely to be detected in all PCR replicates, there can be dropout of other barcode sequences in replicates with lower counts of retained reads. In contrast, read-rare barcode sequences are more likely to be detected in replicates with high retained read counts. We therefore developed a barcode sequence detectability measure, i.e., wtRep, to account for differences in relative counts of retained reads across PCR replicates, by weighting PCR replicates on the basis of their total retained read count relative to the total retained read count across all eight PCR replicates, on a per-sample basis. For example, if a barcode sequence was only detected in replicates 1 and 3, then the wtRep would be as shown in Eq. 1

$$\text{wtRep}=\frac{\Sigma \;\text{retained read count}(\text{rep}.1)+\Sigma \;\text{retained read count}(\text{rep}.3)}{\Sigma \;\text{retained read count}(\text{reps}.1-8)}$$ (1)

where Σ retained read count(rep. ) is the sum of the retained read counts across all the barcode sequences detected in that replicate.

If a PCR replicate were not represented in the retained read data, then it would not contribute to the wtRep score. A limitation of the wtRep score is that it will overrepresent detections in samples or negative controls with few barcode sequences and/or detections. For this reason, we only applied wtRep for a sample if the average proportion of replicates across the sample was ≥0.33 and there were ≥10 barcode sequences present after filtering. For samples that failed this threshold, we used a standard proportion of PCR replicates as a measure of detectability (e.g., 0.25 for the above example).

We further explored the quality of our sedaDNA data by examining four measures. For each sample, we calculated the (i) total count of raw reads (summed across PCR replicates), (ii) mean barcode sequence length (in base pairs) across all retained barcode sequences, (iii) wtRep (see above) across all final barcode sequences, and (iv) proportion of raw reads assigned to terrestrial plant taxa. We compared each of these four measures to both taxonomic richness (Hill-N0) and/or time.

### Nutrient index calculation

We generated a nutrient index for each lake catchment. This new index is derived from the sum of the phosphorus (P), potassium (K), and calcium (Ca) content of the bedrocks modified by a measure of weatherability, in this case, the extended Mohs’ hardness of the least resistant major mineral in the rock type (*H*_min_) (see Eq. 2 and dataset S1). The natural logarithm of Ca content was used as this has been shown to have a strong relationship with pH, which is critical to the availability of nutrients, especially P (*13*)

$$\text{Nutrient index}\;(\text{NI})=\frac{\left(P+K+\text{ln}(\text{Ca})\right)}{H_{\text{min}}}$$ (2)

where P, K, and Ca are total phosphorus, potassium, and calcium in parts per million and *H*_min_ is the hardness of the most easily weathered principal mineral in the local bedrock. We compiled elemental values from bedrock and superficial geology maps from the Geological Survey of Norway online mapping system (www.ngu.no/en) and used the Mohs’ hardness values derived from standard geological tables with additional elemental data from (*11*).

### Numerical and statistical analyses

Using wtRep, we measured taxonomic richness (diversity) based on Hill numbers (N0 and N1) (*69*), as they provide information on both the rare and common taxa within a community (*14*). For each sample, we calculated taxonomic richness as Hill-N0 (total number of observed taxa) and number of abundant taxa as Hill-N1 (see Eq. 3), which is the exponent of the Shannon index (*14*). To evaluate whether differences in sequencing depth between samples affected taxonomic richness estimates, we also calculated rarefied taxonomic richness based on the lowest number of reads assigned to a sample within a lake. We calculated Pearson’s product-moment correlation between rarefied and taxonomic richness (Hill-N0) to evaluate the correspondence between approaches (dataset S8)

$$\text{Hill}-N1=\text{exp}(-\sum p_{i}\text{log}p_{i})$$ (3)

where *p*_i_ is the proportion of each species within a sample.

We used GAMs to evaluate temporal biodiversity changes during the Holocene. GAMs are very efficient at uncovering nonlinear covariate effects and handling nonnormal data that are typical in palaeoecology (*70*). For individual lakes, we treated Hill-N0 and N1 as the response, and median calibrated age of the samples as predictor variables, and used the Poisson distribution with log link. The Hill-N1 was rounded to the nearest whole number before GAM analysis. To account for residual temporal autocorrelation between samples, we also included a continuous-time first-order autoregressive process (CAR1) while fitting GAMs (GAM-CAR1 hereafter) using the “gamm” function following Simpson (*70*). For both GAM and GAM-CAR1 models, the fitted lines are based on the predicted values for 300 points covering the entire range of sample age for each lake. We used a critical value from the *t* distribution to generate a pointwise 95% confidence interval (*70*). We found near identical results for taxonomic richness between GAM and GAM-CAR1 models (fig. S7 and table S2) for all lakes except two shorter cores from Nesservatnet and Sierrvatnet. However, the GAM-CAR1 models provided a more reasonable fit to the data and were therefore included in the main taxonomic richness results for individual lakes (see Fig. 4). We also calculated the accumulated count of taxa through time for each lake (accumulated richness). To evaluate the temporal pattern of taxonomic richness at the regional scale, we fitted a GAMM with lakes as random factor along with a CAR1 process (GAMM-CAR1 hereafter). The model predictions and confidence intervals were calculated as above (see Fig. 5A).

We next evaluated the regional species pool, which we define as the accumulated count of taxa at the regional scale (all 10 lakes combined) from the oldest to the youngest samples across the Holocene. We also calculated the regional species pool for six subsets of the data to assess the impact of data selection. We used (i) two datasets that only contained taxa with greater than two or three occurrences, respectively (see Fig. 5B); (ii) three datasets that used more conservative minimum MAQ score thresholds for sample retention (see fig. S6A); and (iii) a dataset with the two temporally short cores excluded (see fig. S6B). Each of these subsets was compared to the full dataset in their respective figures. Next, we evaluated the rate of taxon accumulation by fitting linear models with accumulated count from the full dataset as the response and years since the first sample as the predictor. Both the response and predictors were log-transformed before regression analysis following Adler and Lauenroth (*71*) (see Fig. 5C).

We assessed the relationship between the regional species pool and mean taxonomic richness. To minimize the effect of extinction on pool size through time, we also estimated the regional species pool as the cumulative count of taxa recorded within 500-year time bins (see Fig. 6). We first binned our samples to the nearest 500 years and estimated the probability of a taxon occurring within each bin based on its co-occurrence with other taxa (*72*, *73*), using the “ThresholdBeal” method as implemented in the DarkDiv (*73*) package and with the minimum probability estimated for samples within a bin as the threshold to generate binary presence/absence data for the regional species pool per 500-year time bin. We then randomly subsampled each regional species pool bin without replacement 1000 times and calculated the mean and SD of accumulated taxa for each 500-year bin using the “accumcomp” function and “random” method as implemented in the BiodiversityR package (*74*). Last, to account for uneven sample size in each time bin, we extracted the regional species pool estimates based on five samples (minimum number of samples in a bin) from each 500-year bin as a measure of standardized regional species pool (see Fig. 6). We evaluated the temporal trend of regional species pool development per 500-year time bin by fitting the linear model with a second-order polynomial term as this provided a better fit (*R*^2^_adj_ = 0.86, *F*_2,20_ = 66.07, *P* < 0.0001) to the data compared to a linear model without a polynomial term (*R*^2^_adj_ = 0.79, *F*_1,21_ = 81.7, *P* < 0.0001). We fitted linear models by considering mean taxonomic richness within a 500-year bin as the response and estimated regional species pool based on five samples per bin as the predictor variable to test whether taxonomic richness is correlated to the regional species pool.

We next examined the relationship of climate and nutrient availability to our taxonomic richness estimates. For climate, we used oxygen isotope (δ^18^O) values from the NGRIP (*35*) as a proxy for temperature. This has limitations as a regional record for northern Fennoscandia because of geographic distance, but its advantage is that it is independent of vegetation-based reconstructions and it covers the whole period of interest. It shows similar trends in annual temperature as both proxy-based and simulated reconstructions for the northern North Atlantic region (*75*). Because summer temperature and growing degree days (GDD) are strong drivers of vegetation response to climate in the north, we note that Holocene GDD sums probably remained higher than present through much of the Holocene, as patterns of both seasonality and season length changed (*75*). The Early Holocene temperature change was steeper than the Middle and Late Holocene, and we expect the taxonomic richness pattern to differ among those periods. Thus, we assigned δ^18^O data of 50-year resolution to the nearest age estimate of samples and split diversity estimates and δ^18^O values into three periods (Early, 11.7 to 8.3 ka; Middle, 8.3 to 4.25 ka; and Late Holocene, 4.25 to 0.0 ka) following Walker *et al*. (*76*). For nutrient availability, we used the nutrient index generated for each lake catchment. We used a linear mixed effects (LME) model to assess the impact of regional climate and nutrient availability on the regional trend of taxonomic richness during the three periods of the Holocene as such a model provided reasonable fit to our data with no evidence for a residual pattern through time (fig. S8). δ^18^O and nutrient index values, and their interactions with the Holocene periods, were the fixed effect, log-transformed taxonomic richness was used as the response, and lake was set as the random effect while fitting the LME model. We also included sample age as a random slope effect to account for different temporal rates of changes in taxonomic richness among lakes.

Unless otherwise stated, all analyses were performed using the vegan package in R (*59*). The library mgcv (*77*) was used for GAM and GAMM, with the “correlation = corCAR1 ()” command used to introduce the CAR1 term, and lme4 (*78*) for LME model building.

## SUPPLEMENTARY MATERIALS

Supplementary material for this article is available at http://advances.sciencemag.org/cgi/content/full/7/31/eabf9557/DC1

## Appendix

View/request a protocol for this paper from *Bio-protocol*.
